# Diabetes Disparities In Illinois

**DOI:** 10.5888/pcd16.180154

**Published:** 2019-02-14

**Authors:** Taylor Jansen, Liliana Aguayo, James Whitacre, Julie Bobitt, Laura Payne, Andiara Schwingel

**Affiliations:** 1 University of Massachusetts Boston, Department of Gerontology, Boston, Massachusetts; 2 Northwestern University Feinberg School of Medicine, Department of Preventive Medicine, Chicago, Illinois; 3 Stanley Manne Children’s Research Institute, Mary Ann & J. Milburn Smith Child Health Research, Outreach, and Advocacy Center, Chicago, Illinois; 4 Carnegie Museum of Natural History, Powdermill Nature Reserve, Rector, Pennsylvania; 5 University of Illinois at Urbana-Champaign, College of Applied Health Sciences, Champaign, Illinois

**Figure:**
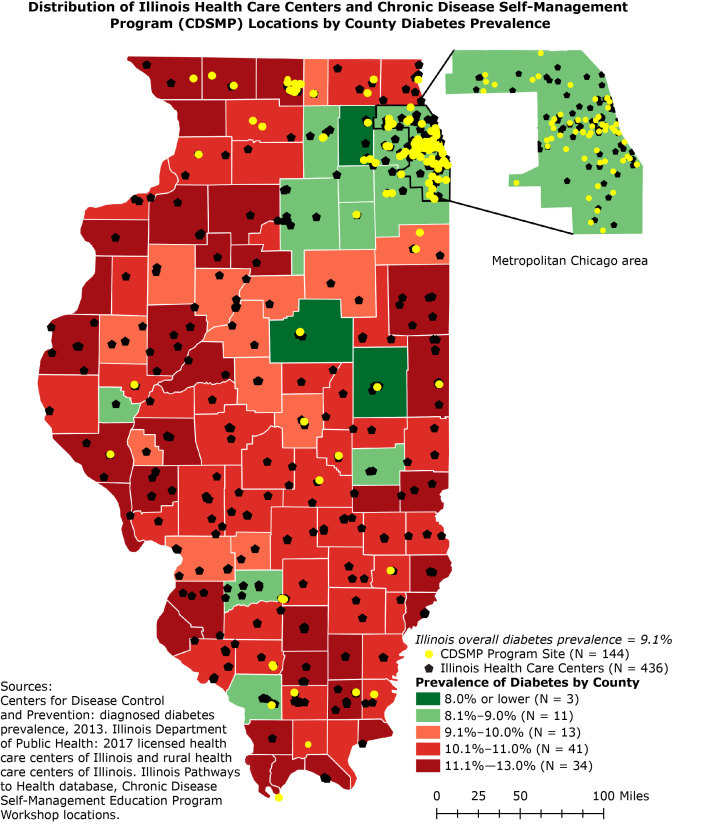
The map depicts the availability of Chronic Disease Self-Management Program workshops and health care centers in Illinois in relation to diabetes prevalence in each county. County-level diabetes prevalence ranges from 7% to 13%, whereas Illinois diabetes prevalence is 9.1%. Green counties have prevalences below the state rate of 9.1%, and red counties have prevalences at 9.1% or higher. Darker colors represent the lowest and highest rates of diabetes prevalence in Illinois.

## Background

Currently, 30.3 million people have diabetes in the United States (1). In 2015, the prevalence of diabetes in Illinois was approximately 9.1% (1). Access to health care centers and health promotion programs are essential in managing diabetes (2). Rural areas are often underserved because they have fewer health care centers (eg, general hospitals, critical access hospitals, rural health centers) (2). Consequently, rural communities have limited opportunities to participate in disease prevention and control programs (2). To address these health disparities, the Administration for Community Living has funded the Illinois Pathways to Health (3) initiative since 2015 to implement evidence-based Chronic Disease Self-Management Programs (CDSMPs) in geographically underserved areas in Illinois. CDSMPs are evidence-based programs recommended by the Centers for Disease Control and Prevention (CDC) to address various chronic diseases (eg, diabetes, heart disease, hypertension, arthritis) (1,2,4,5). Trained facilitators teach participants techniques to manage medicines, improve health literacy, and promote physical activity and nutrition (1,2,4,5). Participation in CDSMPs has been linked to better health outcomes among participants with chronic diseases (2,4,5). Yet, evaluations of program implementation revealed that CDSMPs are most often delivered in urban areas, failing to reach rural counties (2). This map shows geographic differences in diabetes prevalence across Illinois and the associations between diabetes needs and the availability of health care centers and CDSMPs.

## Data Sources and Map Logistics

We obtained data displayed in the map from 3 secondary databases and from Illinois Pathways to Health (3), a program coordinated by the research team. Geographic information for the 2017 licensed health care centers of Illinois and rural health care centers of Illinois was retrieved from the Illinois Department of Public Health data portal (6). These Excel (Microsoft Corp) files were combined and represented on the map as Illinois health care centers. Prevalence of diabetes was retrieved from the CDC’s diagnosed diabetes prevalence county-level indicator (1), which represents estimates of type 1 and type 2 diabetes prevalence in Illinois per county from 2013. Gestational diabetes was not considered (1). Addresses of the CDSMP workshop sites were obtained from our Illinois Pathways to Health database (3). We geocoded health care centers and CDSMP site addresses in Google Earth Pro (Google), transformed into latitude and longitude points, and then plotted them in ArcMap 10.5.1 (Esri, Inc). The numbers of health care centers and CDSMP workshops in each county were calculated into counts in ArcMap by using a spatial attribute join. We used the counts to test the correlations between the number of health care centers and CDSMP workshops with the prevalence of diabetes per county.

Cook County, the metropolitan area of Chicago, was included in the map analysis, but excluded from the statistical testing because it was an outlier. After excluding Cook County, the Shapiro–Wilks test was used to test for normality in the distribution. The findings showed that none of our variables had a normal distribution (prevalence of diabetes *P* = .03, number of workshops *P* < .001, and number of health care centers *P* < .001). Therefore, the nonparametric Spearman ρ correlation test was used to examine the associations between diabetes prevalence with the number of health care centers and with the number of CDSMP workshops offered per county. All statistical tests were conducted using SPSS Statistics 24 (IBM, Inc).

## Highlights

Although the average diabetes prevalence by county in Illinois is 9.1%, diabetes prevalence by county ranged from 7% to 13%. The map represents each county’s diabetes prevalence in 5 intervals of above or below and equal to the state rate of 9.1%, (eg, 8.0% or lower, 8.1%–9.0%, 9.1%–10.0%, 10.1%–11.0%, and 11.1% or higher).

After excluding Cook County, the metropolitan area of Chicago, the number of health care centers per county ranged from 1 to 12 and the number of CDSMP workshops per county ranged from 0 to 10. Cook County had 66 health care centers and 80 CDSMP workshops. A significant negative correlation was found between diabetes prevalence and CDSMP workshops offered per county (*r*_s_ [98] = −0.242; *P* = .02). However, a significant association was not found between the number of health care centers per county and diabetes prevalence (*r*_s_ [98] = −0.001; *P* = .99). Therefore, our findings are that counties with high diabetes prevalence were more likely to have lower access to CDSMP workshops than other counties, while counties with low diabetes prevalence had higher access to CDSMP workshops than other counties.

By neglecting areas with high diabetes prevalence, the allocation of the CDSMP workshops may have spatially widened established health inequalities between rural and urban communities.

## Action

This map highlights the need to provide areas that have high diabetes prevalence with access to health care services and chronic disease programs such as CDSMPs. Reaching underserved populations is a problem that needs to be addressed through intentional planning and collaboration among policy makers and communities (2). To facilitate growth of programs in underserved areas, efforts for diversifying community partners will be needed (2). Future efforts should aim to engage nonclinical health partners (eg, senior centers/Area Agencies on Aging, churches, schools, extension offices, recreation centers) in the implementation of CDSMPs (2). Spatial inequalities should be examined when allocating chronic disease programs to avoid increasing the diabetes disparities between well-served and underserved areas in Illinois.

Our study is limited to examining the availability and access of CDSMP workshops rather than inferring that the presence alone lessens the burden of diabetes. Another consideration is that some programs may be less diabetes-specific than others, and therefore, less likely to reduce the prevalence of diabetes. Although diabetes estimates in the study used data on both type 1 and type 2 diabetes diagnoses, type 2 diabetes accounts for about 90% to 95% of diagnosed diabetes cases in adults (1), and we believe this trend was translated in our sample.

This map can be used to inform Illinois policy makers where the needs are being met and where additional resources, like CDSMPs, would be beneficial.
